# Association Between Interoception and Emotion Regulation in Individuals With Alcohol Use Disorder

**DOI:** 10.3389/fpsyt.2019.01028

**Published:** 2020-02-03

**Authors:** Andrzej Jakubczyk, Elisa M. Trucco, Anna Klimkiewicz, Jakub Skrzeszewski, Hubert Suszek, Justyna Zaorska, Malwina Nowakowska, Aneta Michalska, Marcin Wojnar, Maciej Kopera

**Affiliations:** ^1^ Department of Psychiatry, Medical University of Warsaw, Warsaw, Poland; ^2^ Department of Psychology and the Center for Children and Families, Florida International University, Miami, FL, United States; ^3^ Department of Psychiatry, Addiction Center, University of Michigan, Ann Arbor, MI, United States; ^4^ Department of Psychology, University of Warsaw, Warsaw, Poland

**Keywords:** interoceptive sensibility, interoceptive accuracy, emotion regulation, alcohol use disorder, moderation

## Abstract

**Introduction:**

Sensing body-related information includes interoceptive sensibility (the tendency to focus on internal body sensations) and accuracy (precision in perceiving real internal processes). Interoception and emotion regulation have both been linked to alcohol use disorder (AUD). However, the association between these factors have not been investigated within a clinical group of individuals with AUD.

**Objectives:**

The current study examines associations between emotion regulation and interoceptive accuracy and sensibility among individuals with AUD and healthy controls (HCs).

**Methods:**

The sample comprised 165 individuals meeting criteria for AUD and 110 HCs. Interoceptive sensibility was assessed with a self-report measure (the Private Body Consciousness subscale) and interoceptive accuracy – with a behavioral measure (the Schandry test). Emotion regulation domains: non-acceptance of negative emotions, inability to engage in goal-directed behaviors when experiencing negative emotions, difficulties controlling impulsive behaviors when experiencing negative emotions, limited access to effective emotion regulation strategies, and lack of own emotional awareness and clarity were assessed with the Difficulties in Emotion Regulation Scale (DERS). Associations between interoception and emotion regulation were assessed while controlling for sleep problems, depressive symptoms, age, and sex.

**Results:**

Higher interoceptive accuracy was negatively associated with DERS subscale of non-acceptance of negative emotions in the AUD group (but not in the HC group). Higher interoceptive sensibility was significantly associated with problems in controlling impulsive behaviors when experiencing negative emotions. This association was moderated by symptoms of AUD. Higher interoceptive sensibility was associated with higher emotional awareness, but only in the HC group.

**Conclusions:**

Individuals with AUD who are more interoceptively accurate may be more effective in regulating their emotions. On the other hand, individuals with AUD who are more interoceptively sensible, may have problems with controlling their behaviors while experiencing negative emotional states.

## Introduction

The way an individual receives, processes, and integrates body-relevant signals with external stimuli (such as drugs) contributes to the degree to which he/she approaches or avoids behaviors. Interoception reflects how one perceives stimuli from the body, including temperature, pain, heart rate, and muscle sensations (1). Sensing body-related information includes interoceptive sensibility (the tendency to focus on internal body sensations) and accuracy (precision in perceiving real internal processes measured behaviorally, e.g., heartbeat perception). Prior work with clinical populations (2–5), including individuals with alcohol use disorder (AUD) (6) demonstrated that interoceptive sensibility is not associated with accuracy. A specific phenomenon that is related to correspondence between interoceptive accuracy and sensibility is interoceptive awareness (defined as metacognitive awareness of one’s interoceptive accuracy). The degree to which subjective (interoceptive sensibility) and objective (interoceptive accuracy) facets of interoception overlap within an individual may impact emotional experience (7, 8). Namely, findings indicate that a greater discrepancy between interoceptive accuracy and sensibility is associated with symptoms of high arousal [e.g., sleep problems, anxiety; (3–5)], which may be especially relevant to alcohol use, as alcohol is commonly used to regulate arousal. Consequently, interoception has recently reached increased attention as a factor that may impact both: the development and course of addiction (9).

Interoception may contribute to problematic substance use through the “embodied” experience of substance use or withdrawal (9). It is suggested that the interplay between an individual’s predicted versus actual internal state may modulate approach or avoidance behavior in terms of whether or not to use a substance to enhance or decrease embodied arousal (9). Prior work (10, 11) demonstrated that lower levels of embodied perception of alcohol intoxication (lower interoceptive accuracy) predicted higher risk for AUD. Moreover, alcohol likely impairs interoceptive accuracy (12), characterizing the vicious cycle that maintains the disorder. Importantly, neural areas (e.g., the insula) related to interoception may also contribute to craving states (13). On the other hand, adolescents diagnosed with substance use disorder demonstrated altered insula response to pleasant interoceptive stimuli (14). Prior work indicates that individuals with AUD are characterized by lower interoceptive accuracy and higher sensibility in comparison to healthy controls (6). In this sample, interoceptive sensibility was also associated with high levels of anxiety, depression, and more severe sleep problems. This suggests that individuals with AUD may experience higher interoceptive sensibility, higher arousal (i.e., tension and sleep problems), and an approach attitude towards alcohol as a compensatory strategy for poor interoceptive accuracy. Importantly, therapeutic interventions aimed at improving interoception awareness skills, such as mindfulness-based approaches, were shown to be effective in women’s substance abuse treatment (15). In addition to general interoceptive abilities, sensitivity to one specific type of interoceptive sensation – pain, was shown to be an important factor contributing to the development and course of AUD (6, 16, 17). Interestingly, it was shown that consuming alcohol may differentially influence pain sensations in individuals with AUD and healthy controls. Specifically, alcohol may alleviate the experience of pain in healthy controls but may intensify the experience of pain in patients with AUD (16).

Another construct that is relevant to problematic alcohol use is emotion regulation. Emotional dysregulation is a multi-faceted construct involving: a lack of awareness, understanding, and acceptance of emotions; an inability to control behaviors when experiencing emotional distress; a lack of access to adaptive strategies for modulating the duration and/or intensity of aversive emotional experiences; and an unwillingness to experience emotional distress (18). Impairment in emotion regulation was shown to be a strong motivator for alcohol use and a core emotional disturbance among individuals with AUD (19). Moreover, poor emotion regulation was associated with increased reports of experiencing more severe physical pain (20) and poorer outcomes (21, 22) among individuals with AUD. In addition, poor emotion regulation is commonly accompanied by depressive symptoms (23) and sleep problems (24), which are important factors contributing to the development of AUD and poor treatment outcomes (25, 26). Importantly, it was shown that distinct facets of emotion dysregulation (e.g., lack of awareness, clarity, lack of behavioral control in emotional situations) may manifest differently across various clinical samples (27).

There is growing evidence that interoceptive responses are associated with immediate discrete emotions (7, 28); however, individuals may differ in terms of their ability to recognize their emotional states. Alexithymia is considered a deficit in the cognitive processing of emotion and an impairment in the mental representation of emotions (as feelings), which limits the capacity to regulate emotions through cognitive processes. Importantly, prior work indicates that alexithymia is present in 50% to 78% of individuals with an AUD (29) in comparison to 10% of individuals in the general population (30).

Grounded in neurobiological studies, Craig (1) posited that interoception should be redefined to reflect the physiological condition of the entire body, not just the internal organs, as the perception of the body’s response to different stimuli and its impact on one’s emotional experience. It is suggested that greater accuracy in sensing one’s bodily state may facilitate the regulation of emotional responses, as ongoing bodily changes can be detected more accurately (1). Moreover, greater interoceptive accuracy might constitute a positive precondition for effective self-regulation of emotionally-driven behavior in healthy individuals (31). The observation that the size and complexity of the human interoceptive system may differentiate humans from sub-human primates emphasizes the significance of studies on associations between emotions and interoception (1). Similarly, difficulties in emotion regulation were associated with deficits in interoceptive awareness in individuals with moderate and severe obesity (32), healthy volunteers (33) individuals with autism (8) or individuals with borderline personality (34).

However, to the best of our knowledge, associations between emotion regulation and interoception have not been investigated among individuals with AUD. This could have utility given the significance of both interoception and emotion regulation within this clinical sample (6, 20). Moreover, evidence for a common neurobiological underpinning for processes associated with interoception, emotion regulation, and craving lend support to a plausible connection between these constructs. It was shown that insula activity represents the interoceptive effects of drug taking, making this information available to conscious awareness, memory, and executive functions. Therefore, in situations associated with emotional arousal, a history of positive interoceptive (and emotional) experience with alcohol may elicit craving and encourage alcohol consumption in order to repeat previously experienced somatic and emotional relief (35). The current study analyzes associations between emotion regulation and interoception in individuals with AUD. Given possible discrepancies between different dimensions of interoception, as well as concerns regarding the validity of various methods for assessing interoception (36), the current study adopts both behavioral and questionnaire assessments to measure interoceptive accuracy and sensibility. Moreover, given that previous studies demonstrate that specific interoceptive sensations may be processed differently across AUD and non-AUD samples (16), we also investigated whether the association between interoception and emotion regulation is moderated by AUD status.

It was hypothesized that better emotion regulation would be associated with higher interoceptive accuracy and lower interoceptive sensibility among individuals with AUD. In addition, given that emotion regulation is a complex, multidimensional construct, it was hypothesized that distinct facets of emotion regulation may have different associations with interoception when comparing individuals with AUD and those without AUD.

## Materials and Methods

### Participants and Procedures

#### Individuals With Alcohol Use Disorder

Participants for this study comprised 165 individuals 18 years or older that were admitted to an eight-week, drug-free, abstinence-based, inpatient alcohol treatment program in Warsaw, Poland. For the current study, only participants meeting criteria for AUD were recruited. Study procedures were performed during the first two weeks after treatment admission. Due to an overrepresentation of men in Polish substance use treatment programs, the sample was composed primarily of White men (88.1%). On average, participants were 44.0 ± 11.2 years of age. The average age for the onset of alcohol drinking problems was 25.7 ± 9.6 years of age. The average duration of the last drinking period (as reported by participants) was 69.5 ± 196.6 (range = 1–1460) days. The maximum amount of daily alcohol consumption during the last drinking period was 285.8 ± 200.3 standard units (where 1 standard unit = 10 grams of 100% ethanol according to European calculations). Lastly, participants reported an average of 49.2 ± 45.1 days of abstinence prior to the day of their assessment. Thus, participants for the current project reflect individuals with severe symptoms and consequences of AUD, but without acute symptoms of withdrawal.

The diagnosis of AUD was based on the International Classification of Diseases and Related Health Problems 10th Revision (37) during their admission to the addiction treatment center. Then, the diagnosis was confirmed by investigators using the MINI International Neuropsychiatric Interview (38) during the study assessment procedures. Individuals were ineligible for the treatment program if they had a clinically significant cognitive deficit (<25 on the Mini-Mental State Examination) (39) or met any of the following criteria: a history of psychosis, co-occurring psychiatric disorders requiring current medication, or the presence of acute alcohol withdrawal symptoms.

#### Healthy Controls

A healthy control (HC) group was included in the study design to confirm differences between individuals with and without AUD symptoms in terms of interoception and emotion regulation. HCs were also included to investigate whether associations between emotion regulation and interoception differ between individuals with AUD.

HCs (n = 110; 74.5% men) included individuals presenting to a general practitioner for treatment of an infection, for a prophylactic examination, or for medical advice. The primary exclusion criterion for the HC group was presence of AUD symptoms or harmful alcohol use as assessed by the Alcohol Use Disorders Identification Test (AUDIT) (40). Other exclusion criteria were similar to those for the AUD group. On average, HCs were 40.6±8.1 years of age. A comparison across the two groups revealed that individuals with AUD were significantly older (p = 0.006, df = 1, F = 7.65) and more likely to be male (χ^2^^=^ 8.2, df = 1, p = 0.004). Therefore, age and sex were used as control variables in all subsequent analyses.

This study was conducted in accordance with the ethical principles described in the Declaration of Helsinki in 1964 and received approval from the Bioethics Committee of the institution where the study took place.

### Measures

#### Psychiatric Comorbidity

Comorbidity was assessed with the Polish version of the MINI International Neuropsychiatric Interview (41). The Polish version of Brief Symptom Inventory (BSI) (42) and the Athens Insomnia Scale (43) were utilized to assess depressive symptoms and sleep problems, respectively. Both have been associated with interoception and emotion regulation (3, 4, 44) and therefore were taken into consideration as control variables in the study design.

#### Interoceptive Accuracy

The mental tracking task developed by Schandry (45) was administered by instructing participants to silently count their heartbeats in trials of different lengths (25s, 35s, and 45s). Interoceptive accuracy was calculated using the following formula:

1/3∑(1−(|actual heartbeats−reported heartbeats|)/actual heartbeats).

Perfect correspondence between the reported and actual heartbeats is equal to one. The heartbeat perception test was available for 86 individuals with AUD (52%) and 104 (94%) HCs. There were no significant differences in psychopathological characteristics or emotion regulation scales between those individuals with and without available Schandry test data.

#### Interoceptive Sensibility

The Private Body Consciousness subscale (PBCS) (46) from the Body Consciousness Questionnaire consists of 5 items reflecting the tendency to focus on internal body sensations. Specifically, it assesses awareness of heart beating, internal bodily tensions, hunger, dry mouth/throat, and body temperature (e.g., “I am sensitive to internal body tensions’’). Higher scores indicate greater interoceptive sensibility. Analyses were performed for all participants. Results were similar among individuals with available Schandry test data. Data was available for 141 (85%) individuals with AUD and 107 (97%) healthy controls. Cronbach α value for this scale was 0.71.

#### Emotion Regulation

The Polish version of Difficulties in Emotion Regulation Scale (DERS) (19, 47) was used to assess emotion dysregulation across six domains: non-acceptance of negative emotions (DERS_non-acceptance_), inability to engage in goal-directed behaviors when experiencing negative emotions (DERS_goal_), difficulties controlling impulsive behaviors when experiencing negative emotions (DERS_impulse_), limited access to effective emotion regulation strategies (DERS_strategies_), and lack of own emotional awareness (DERS_awareness_) and clarity (DERS_clarity_). Higher scores on these subscales indicate *worse* emotion regulation. Cronbach α values were 0.92 for total DERS score, 0.82 for DERS_non-acceptance_, 0.80 for DERS_goal_, 0.83 for DERS_impulse_, 0.76 for DERS_awareness_, 0.84 for DERS_strategies_, and 0.73 for DERS_clarity_.

### Statistical Analyses

First, an analysis of variance (ANOVA) was used to compare the two groups (AUD and HCs) across the emotion dysregulation subscales and the two measures of interoception. Second, correlations between interoceptive accuracy (Schandry score) and sensibility (PBCS score) and all subscales of emotion regulation (DERS) were conducted. Emotion regulation was treated as a main dependent variable, and therefore, in subsequent steps, emotion regulation subscales that were correlated with interoception were tested for associations with possible confounders (i.e., severity of depression, sleep problems, characteristics of alcohol drinking). Finally, for all emotion regulation domains (DERS subscales) that were significantly associated with interoception measures, linear regression models were estimated to examine whether other factors were associated with emotion regulation in the AUD group. None of the drinking characteristics (onset of drinking problems, number of consecutive drinking days during the last drinking period, maximum amount of alcohol drunk daily during the last drinking period, length of abstinence prior to the assessment) in the AUD group were significantly associated with emotion regulation subscales. Therefore, these variables were not included in multivariate models.

To compare associations between emotion regulation and interoception across the two groups, similar models were estimated for HCs. Subsequently, to test AUD-status (AUD vs. HC) as a potential moderator of the association between interoception and emotion regulation, Preacher and Hayes’ (2008) PROCESS SPSS macro for moderation with bootstrapping (5000 resamples with replacement) was applied when significant albeit opposing directions of correlations between emotion regulation and interoception were observed across the two groups in linear regression models. For these analyses non-standardized coefficients are reported. Moderation was considered a post-hoc analysis in this study, as no specific hypotheses regarding the differences across groups could be formulated based on the lack of prior work comparing AUD samples to healthy controls.

## Results

### Comparison Between Groups

Individuals with AUD scored significantly higher on all domains of emotional dysregulation. In addition, individuals with AUD scored significantly higher on the interoceptive sensibility scale (PBCS), but significantly lower on the behavioral measure of interoceptive accuracy (Schandry score) (for details see **Table 1**).

**Table 1:** Comparison between individuals with alcohol use disorder (AUD) and healthy controls (HC).

	AUD (mean±SD)	HC (mean±SD)	p
DERS_total_	90.3±19.3	70.0±14.5	<0.0005
DERS_non-acceptance_	14.2±4.8	11.7±4.28	<0.0005
DERS_impulse_	13.8±4.4	9.8±3.3	<0.0005
DERS_awareness_	17.1±4.9	14.6±4.2	<0.0005
DERS_clarity_	11.9±3.6	8.8±2.9	<0.0005
DERS_strategy_	19.2±6.1	14.1±3.3	<0.0005
DERS_goals_	14.1±3.9	10.9±3.2	<0.0005
PBCS	17.3±4.1	14.8±4.1	<0.0005
Schandry score	0.61±0.16	0.72±0.08	<0.0005
Depressive symptoms (BIS_dep_)	1.03±0.7	0.2±0.3	<0.0005
Sleep problems (AIS)	7.5±4.4	3.7±3.1	<0.0005

SD, standard deviation; BSI, Brief Symptom Inventory; AIS, Athens Insomnia Scale; PBCS, Private Body Consciousness subscale (interoceptive sensibility); anx, anxiety symptoms; CI, confidence interval; DERS, Difficulties in Emotion Regulation Scale; DERS_impulse_, difficulties controlling impulsive behaviors when experiencing negative emotions; DERS_awareness_, lack of own emotional awareness; DERS_non-acceptance_, non-acceptance of negative emotions; DERS_goal_, inability to engage in goal-directed behaviors when experiencing negative emotions; DERS_strategies_, limited access to effective emotion regulation strategies; DERS_clarity_, lack of own emotional clarity; AUD, alcohol use disorder; HC, healthy controls.

There were no significant correlations between interoceptive accuracy and sensibility in either of the groups (*r* = 0.12, *p* = 0.23 in HCs; *r* = 0.12, *p* = 0.31 in individuals with AUD).

### Interoceptive Accuracy

Within the AUD group, interoceptive accuracy (higher Scandry score) was negatively correlated with DERS_non-acceptance_ score (DERS_non-acceptance_: non-acceptance of negative emotions; *r* = - 0.30, *p* = 0.006). There were no other significant correlations observed between the other DERS subscales and interoceptive accuracy. Non-acceptance of negative emotions was also positively correlated with depressive symptoms (BSI_dep_) (*r* = 0.32, *p* < 0.0005). Therefore, the Schandry score, depressive symptoms (BSI_dep_), and control variables (age and sex) were added to the linear regression analyses predicting non-acceptance of negative emotions across both groups. Findings indicate that interoceptive accuracy was negatively associated with non-acceptance of negative emotions in the AUD group (but not in the HC group), when controlling for sex, age, and depressive symptoms (see **Table 2**).

**Table 2:** Linear regression analysis for the predictors of DERS_non-acceptance_ in individuals with alcohol use disorder (N = 86) and healthy controls (N = 104).

	AUD			HC		
	**Beta**	**95%CI**	***p***	**Beta**	**95%CI**	***p***
**Sex**	−0.14	−5.2 to 0.96	0.17	0.035	−0.01 to 16.3	0.71
**Age**	0.03	−0.09 to 0.13	0.74	0.051	−0.07 to 0.12	0.59
**Schandry score**	−**0.31**	−**16.0 (**−**3.2)**	**0.004**	0.036	−7.7 to 11.35	0.7
**Depressive symptoms (BSI_dep_)**	**0.32**	**0.75**–**3.46**	**0.003**	**0.33**	**1.64**–**6.34**	**0.001**
	Rsquare = 0.203 Corrected Rsquare = 0.161 *p* < 0.002			Rsquare = 0.111 Corrected Rsquare = 0.075 *p* = 0.019		

BSI_dep_, Brief Symptom Inventory, severity of depressive symptoms score; AUD, alcohol use disorder; HC, healthy controls, DERS_non-acceptance_, non-acceptance of negative emotions. The bolded texts refer to the p value lower than 0.05.

### Interoceptive Sensibility

Within the AUD group, higher interoceptive sensibility (higher PBCS score) was positively associated with difficulties controlling impulsive behaviors when experiencing negative emotions (DERSE_impulse_; *r* = 0.19, *p* = 0.026) and negatively correlated with DERS_awareness_ score (DERS_awareness_: lack of own emotional awareness; *r* = - 0.19, *p* = 0.024).

Additionally, difficulties controlling impulsive behaviors when experiencing negative emotions (DERS_impulse_) were associated with more severe depressive symptoms (BSI_dep_; *r* = 0.46, *p* < 0.0005) and sleep problems (AIS; *r* = 0.17, *p* = 0.037). Therefore, interoceptive sensibility, severity of sleep problems, and depressive symptoms (BSI_dep_), together with control variables (age and sex) were added to the linear regression analyses predicting difficulties controlling impulsive behaviors when experiencing negative emotions_._ Findings indicate that interoceptive sensibility was significantly associated with controlling impulsive behaviors when experiencing negative emotions across both groups, even when controlling for sex, age, and depressive symptoms (see **Table 3**).

**Table 3:** Linear regression analysis for the predictors of DERS_impulse_ in individuals with alcohol use disorder (N = 141) and healthy controls (N = 107).

	AUD			HC		
	**Beta**	**95%CI**	***p***	**Beta**	**95%CI**	***p***
**Sex**	0.05	4.2–13.8	0.5	−0.01	7.9–15.7	0.87
**Age**	−0.07	−1.2 to 2.5	0.34	−0.06	−0.1 to 0.04	0.45
**PBCS**	**0.16**	**0.01**–**0.3**	**0.039**	−**1.99**	−**0.3-(**−**0.02)**	**0.029**
**Depressive symptoms (BSI_dep_)**	**0.49**	**2.0**–**3.9**	**<0.0005**	**0.5**	**3.2**–**6.7**	**<0.0005**
**Sleep problems (AIS)**	−0.09	−0.25 to 0.1	0.28	0.18	−0.001 to 0.42	0.051
	Rsquare = 0.274 Corrected Rsquare = 0.247 *p* < 0.0005			Rsquare = 0.311 Corrected Rsquare = 0.277 *p* < 0.0005		

BSI, Brief Symptom Inventory; AIS, Athens Insomnia Scale; PBCS, Private Body Consciousness Subscale (interoceptive sensibility); dep, depressive symptoms; CI, confidence interval; AUD, alcohol use disorder; HC, healthy controls; DERS_impulse_, difficulties controlling impulsive behaviors when experiencing negative emotions. The bolded texts refer to p values lower than 0.05.

**Figure 1:**
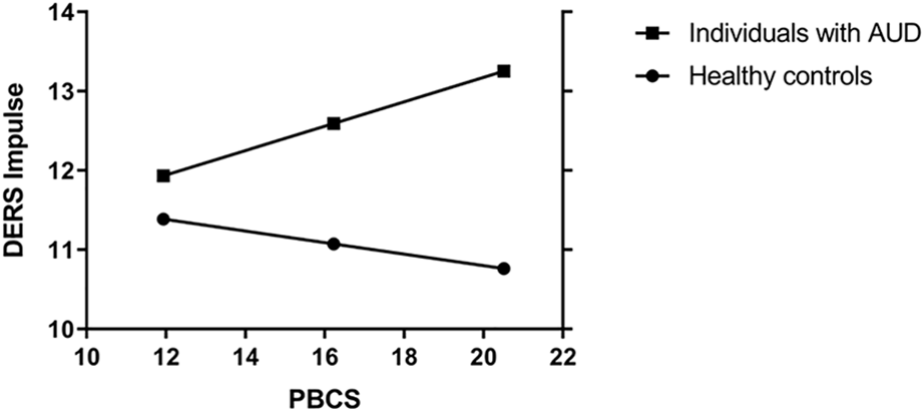
Moderation on the association betweeen interoceptive sensibility on DERS_impulse_ with biological sex, age, depressie symptoms and sleep problems as covariates. PBCS, Private Body Conciousness Subscale; DERS_impulse_, difficulties controlling impulsive behaviors when experiencing negative emotions; AUD, alcohol use disorder.

However, in the AUD group, higher interoceptive sensibility (PBCS) was associated with a *higher* DERS_impulse_ score (more difficulties controlling impulsive behaviors when experiencing negative emotions), while in the HC group, higher interoceptive sensibility was significantly associated with a *lower* DERS score (less severe difficulties controlling impulsive behaviors when experiencing negative emotions). Additional, analyses were conducted to examine whether these opposing findings could be due to the role of AUD-status as a potential moderator of the association between difficulties controlling impulsive behaviors when experiencing negative emotions and interoceptive sensibility while controlling for biological sex, age, and depressive and sleep problem severity. The model explained 41% of the variance in controlling impulsive behaviors when experiencing negative emotions (*R* ^2^ = 0.41; F [7, 239] = 23.48; *p* < 0.001). A significant interaction was found between AUD status and interoceptive sensibility (*b* = 0.23, CI [0.01–0.44], *p* = 0.04, Δ*R* ^2^ = 0.01). The interaction is presented graphically in **Figure 1**. Findings indicate that the simple slope of interoceptive sensibility was statistically significant in individuals with AUD (*b* = 0.15; CI [0.01–0.30]; *p* = 0.04), but not significant in the HC group (*b* = −0.07; CI [−0.23 to 0.09]; *p* = 0.34). That is, interoceptive sensibility was positively associated with difficulties controlling impulsive behaviors when experiencing negative emotions, but only among individuals with AUD.

A lack of own emotional awareness was not associated with any additional analyzed variables in the AUD group, except for interoceptive sensibility (see above); therefore, only interoceptive sensibility, age, and sex were included in the linear regression model predicting lack of own emotional awareness in the AUD and HC groups. Results indicated that interoceptive sensibility was not significantly associated with a lack of own emotional awareness when controlling for age and sex in the AUD group; however, it was significant in the HC group (see **Table 4**).

**Table 4:** Linear regression analysis for the predictors of DERS_awareness_ in individuals with alcohol use disorder (N = 141) and healthy controls (N = 107).

	AUD			HC		
	**Beta**	**95%CI**	***p***	**Beta**	**95%CI**	***p***
**Sex**	−0.06	−3.3 to 1.5	0.47	−0.004	−1.8 to 1.7	0.97
**Age**	0.1	−0.03 to 0.1	0.25	−0.12	−0.16 to 0.03	0.2
**PBCS**	−0.16	−0.4 to 0.01	0.064	−**0.31**	−**0.5-(**−**0.12)**	**0.001**
	Rsquare = 0.050 Corrected Rsquare = 0.029 p = 0.071			Rsquare = 0.106 Corrected Rsquare = 0.079 p = 0.009		

AUD, alcohol use disorder; HC, healthy controls; PBCS, Private Body Consciousness Subscale (interoceptive sensibility); DERS_awareness_, lack of own emotional awareness. The bolded texts refer to *p* values lower than 0.05.

## Discussion

As expected, individuals with AUD were characterized by lower interoceptive accuracy and higher interoceptive sensibility in comparison to healthy, non-alcohol-dependent controls. Moreover, also as expected, individuals with AUD scored significantly higher on all subscales of emotion dysregulation compared to HCs, which is consistent with the larger literature (19). A novel finding of our study is that among individuals with AUD, greater interoceptive accuracy was associated with better emotion regulation; specifically, better ability to accept emotional distress/negative emotions. At the same time, in the AUD group, higher interoceptive sensibility was associated with worse emotion regulation; specifically, difficulties in controlling behaviors when experiencing emotional distress. However, this association was moderated by AUD status and was not present among HCs. In addition, among HCs higher interoceptive sensibility was associated with better emotional awareness, while in the AUD group this association was not significant when controlling for other relevant variables. To our knowledge this is the first study to: 1) analyze associations between interoception and emotion regulation among individuals with AUD and 2) compare similar associations to healthy, non-alcohol-dependent controls.

Our findings are consistent with evidence indicating that perceptions of bodily reactions to various stimuli may be a crucial component for mediating emotional experiences. Damasio’s seminal work claimed that emotional feeling awareness is based on the neural representation of bodily cognitions, with ‘somatic markers’ evoking feeling states that influence cognition and behavior (48). Subsequently, based on recent neurobiological findings (namely, work on functional connections between the insula, anterior cingulate cortex, lamina I spinothalamocortical projections, and orbitofrontal cortex, which are responsible for transforming signals from the body into their cortical, conscious representation), Craig (1) postulated that interoception should be redefined as the sense of the physiological condition of the entire body, not just the viscera. Recently, Pistoia and colleagues found that individuals with spinal cord injury (associated with deficits in interoceptive transmission) had difficulties judging their own emotional response to complex scenes eliciting fear and anger. Authors concluded that the proper perception of internal states (interoception) through preserved sensory pathways is essential for experiencing primordial emotions (49). Consistent with our results, Fustos and colleagues demonstrated that awareness of one’s bodily signals may constitute a precondition for effective self-regulation of behavior among healthy individuals (31). Additionally, Schaefer et al. (50) found that improving heartbeat perception in patients with medically unexplained symptoms significantly reduced distress (50). Thus, recent findings support the idea that greater accuracy in recognizing one’s bodily state may facilitate the regulation of emotional responses, as ongoing bodily changes can be detected more accurately. This in turn may help regulate emotional states and lead to the constructive use of energy derived from emotional arousal towards adaptive cognition and action.

The current study expands existing knowledge on the association between emotion regulation and interoception to a group of individuals who are more at risk of using maladaptive methods (i.e., drinking alcohol) to manage negative emotions, and also cope with unpleasant internal somatic (interoceptive) states (19). Those somatic and emotional states may be independent of drinking (e.g., related to stress), but also associated with withdrawal. In our study, better interoceptive accuracy was associated with higher ability to accept unpleasant, negative emotions. This may be an important finding, because it utilizes an objective, behavioral measure, which was found to be related to one’s beliefs about his/her emotion regulation independent of depressive symptoms. As shown in basic comparisons, individuals with AUD are characterized by poor awareness and clarity of emotions. These characteristics may predispose an individual to experience indefinite tension or arousal, which (when not accepted) may lead to impulsive ways of releasing the tension like alcohol drinking (51). Therefore, higher ability to accept these negative emotional states (which as shown in our study may be supported by higher ability to identify and describe somatic states) may plausibly constitute a protective factor against drinking. These observations are consistent with prior work supporting the significant role that alexithymia plays in the etiology of problematic alcohol use. For example, unpleasant arousal typically experienced by individuals with alexithymia has been demonstrated to be linked with maladaptive coping behaviors, including excessive alcohol consumption (52).

Leganes-Fonteneau (53) showed that metacognitive cardiac awareness correlated with higher acuity in the perception of alcohol-induced responses (specifically: light headedness). That is, the greater one’s ability to recognize how well they perceive their internal bodily sensations, the more likely they are to experience the effect of substances on their mental and physical state. This study, although conducted on healthy non-alcohol-dependent volunteers, supports the idea that interoceptive abilities may constitute the basis of perceiving the effects of alcohol, which in turn can act as an interoceptive related cue and (as shown in our study) may represent an additional risk factor involved in the development of AUD. In our study, interoceptive awareness (metacognitive awareness of interoceptive accuracy, e.g. sensibility-accuracy correspondence) was not specifically addressed; however, we did observe a significant sensibility-accuracy discrepancy in AUD individuals, which (according to Leganes-Fonteneau et al., 2019 study) might predispose individuals to lower alcohol sensitivity and subsequently higher alcohol consumption. As mentioned earlier, a common characteristic present among individuals with AUD is alexithymia. Alexithymia has been empirically linked with low interoceptive capabilities (54) thus underscoring the link between bodily and emotional awareness. New data recently revealed that high alexithymia might be a consequence of a multi-domain failure of interoception (55). Similar to interoception, alexithymia has been related to functional, structural, and neurochemical integrity of the anterior insular cortex (56, 57). Accordingly, it is plausible that interoception and alexithymia may jointly influence problematic alcohol use. In addition, Betka and colleagues (58) observed that alexithymia fully mediated the relationship between sensitivity to bodily sensations and alcohol consumption in a normative sample. Interestingly, there is great deal of evidence that supports the predictive ability of measures of alexithymia and interoception on physical and mental health in childhood. For example, high alexithymia and low interoceptive accuracy were associated with obesity, lower physical activity, worse glycemic control, higher levels of anxiety and negative mood in children (59). In contrast, in some studies atypically high interoceptive accuracy was associated with greater psychopathology (e.g., autism, panic disorder) (59). Research on interoception in adolescence is scarce; however, there is converging evidence supporting an association between high alexithymia and more severe psychopathology. Specifically, during adolescence, alexithymia has been associated with anxiety, depression, delinquency, conduct disorder, and binge drinking/AUD as a means to cope with these clinical syndromes (59). In general, the data from developmental studies suggest that atypical (high or low) interoception may contribute to the onset of psychopathology in adolescence leading to an increased risk of subsequent problematic alcohol use. As a consequence, disruption of interoception and alexithymia may further develop as a long-term consequence of heavy consumption of alcohol through its adverse effects on brain structures involved in cognitive regulation of emotional processes (e.g., the amygdala, the anterior cingulate cortex, anterior insula) (60).

We observed that the association between interoceptive sensibility and emotion regulation (specifically, behavioral control while experiencing negative emotions) is moderated by AUD-status. This is a novel and interesting finding suggesting that among individuals with AUD, higher interoceptive sensibility may not represent an adaptive characteristic supporting emotion regulation. On the contrary, it may worsen emotional regulation, plausibly by increasing arousal and leading to impulsive behaviors while experiencing distress. From a neurobiological perspective, the neural circuits involved in processing visceral afferents overlap substantially with those involved in arousal-related processing. Namely, the insula has been found to be responsible for control, salience assessment, and reward processing (9). These connectivity patterns suggest that the insula may be important for translating interoceptive/emotional stimuli into activation of the cognitive control network to implement goal-directed behavior, especially those aimed at decreasing arousal (like drinking alcohol).

Another domain of emotion regulation – emotion awareness – was positively correlated with interoceptive sensibility in the HC group, but not among individuals with AUD. This could have important clinical implications. Namely, there may be utility in not only addressing interoceptive accuracy in treatment, but also targeting emotions that accompany focusing on the body (interoceptive sensibility). Our study shows that among individuals with AUD, not only is there a clear discrepancy between different facets of interoception, but the same domain of interoception (interoceptive sensibility) may be associated differently with emotion regulation depending on AUD-status. Therefore, it is possible that among individuals with AUD, the adaptive link between a greater focus on somatic states and emotion regulation (which we observed in HCs) is disrupted, leading to dysregulation of emotional processes. The mechanisms of this disruption (somatic states from cognition about somatic states, mind from the body) may resemble dissociative processes. These are not thoroughly investigated in AUD; however, prior work supports the possible role of dissociative processes among individuals with AUD as alcohol may be used to separate mental state from body conditions (61). Similarly, the abovementioned dysregulation of somatic and emotional processes may lead to alcohol drinking. This is plausible especially given that alcohol may be used for self-medication purposes to simultaneously manage negative somatic and emotional experiences.

Our study may have important clinical implications given prior work indicating that improving interoceptive accuracy may facilitate emotional skills (15, 50), as well as our results showing that greater interoceptive accuracy is associated with better regulation of emotions. It is possible that therapeutic strategies aimed at interpreting bodily signals may affect emotion regulation, and in turn influence alcohol use. In a study by Price and colleagues (15), interoceptive awareness training through mindful awareness in body-oriented therapy implemented as an adjunct to women’s substance use disorder treatment turned out to be efficient for reducing substance use, but also improved craving and emotion regulation (as assessed by respiratory sinus arrythmia). These findings suggest the potential utility of adopting similar interventions specifically for AUD.

### Limitations

Our study has limitations that should be noted. This is a cross-sectional study in which only participants from an inpatient treatment program for AUD were recruited. These are individuals with a severe course of AUD, severe emotion dysregulation, and negative consequences of drinking. The majority of the sample was men. Thus, our results cannot be generalized to all individuals with AUD. Individuals with AUD were significantly older and more likely to be male in comparison to healthy controls. Although age and sex were used as control variables in all analyses, it is possible that the older age of individuals with AUD may have influenced the results. Older age among individuals with an AUD typically translates to a longer period of harmful drinking, which in turn may affect interoceptive accuracy through the damage of the central and peripheral nervous system. Also, given the low number of women in the AUD sample, it is unclear whether there are any sex differences in terms of the association between emotion regulation and interoception in AUD sample. Previous research showed that women may be generally more interoceptively sensible, but less accurate in comparison to men (62). Although we did not observe such differences in either of our groups it would be informative to conduct such comparisons in future studies on individuals with alcohol use disorder. The Schandry test was only available for 52% of individuals from the AUD group. Also, the validity of the Schandry test as a measure of interoceptive accuracy has recently been questioned since this task may be influenced by beliefs and expectancies about heart rate or the ability to retain a count in working memory (36). However, there is also evidence that results of the Schandry test correspond well with other interoceptive tasks (63). In addition, all measures of emotion regulation were based on self-report. Future studies examining associations between interoception and emotional impulsivity (i.e., positive and negative urgency), as well as behaviorally measured emotion regulation are likely to have utility.

## Conclusions

Individuals with AUD who are more interoceptively accurate may be more effective in regulating their emotions. On the other hand, individuals with AUD who are more interoceptively sensible, may have problems controlling their behaviors while experiencing negative emotional states. These relationships may be specific for individuals with AUD, as they were not present in healthy, non-alcohol-dependent controls.

## Data Availability Statement

The datasets generated for this study are available on request to the corresponding author.

## Ethics Statement

The studies involving human participants were reviewed and approved by Bioethics Committee of Medical University of Warsaw. The patients/participants provided their written informed consent to participate in this study.

## Author Contributions

All authors contributed to the conceptualization and design of the analyses. MK and AJ designed the study and wrote the protocol. AJ, MK, JS, JZ, MN, and AM contributed to the data collection. AJ, MK, ET, and HS took responsibility for conducting analyses. AJ, MK, HS, MW, MN, and JS managed the literature search. AJ and MK wrote the first draft of the manuscript. ET, HS, MW, JZ, and AM provided substantive and conceptual feedback on all drafts. All authors contributed to and have approved the final manuscript.

## Funding

This study was supported by the National Science Centre grant (2017/25/B/HS6/00362; PI: Jakubczyk), the National Institute on Alcohol Abuse and Alcoholism (K08 AA023290; PI Trucco), and the National Institute on Minority Health and Health Disparities (U54 MD012393; Sub-Project ID:5378; Co-PIs Trucco and Matthew Sutherland).

## Conflict of Interest

The authors declare that the research was conducted in the absence of any commercial or financial relationships that could be construed as a potential conflict of interest.
